# A study on the differential protein profiles in liver cells of heat stress rats with and without turpentine treatment

**DOI:** 10.1186/1477-5956-7-1

**Published:** 2009-01-07

**Authors:** Ganapathy Rajaseger, Chin Leong Lim, Lee Koon Wui, Padmanabhan Saravanan, Kai Tang, Ponnampalam Gopalakrishnakone, Yap Eric Pen-huat, Jia Lu, Moochhala M Shabbir

**Affiliations:** 1 Defence Medical & Environmental Research Institute, DSO National Laboratories, 27 Medical Drive, #09-01 Kent Ridge117510, Singapore; 2 Department of Anatomy (MD10), Venom and Toxin Research Programme, Yong Loo Lin School of Medicine, National University of Singapore, Lower Kent Ridge Road 117597, Singapore; 3 School of Biological Sciences, Nanyang Technological University, 60 Nanyang Drive 637551, Singapore

## Abstract

**Background:**

Heat stress (HS) and related illnesses are a major concern in military, sports, and fire brigadiers. HS results in physiologic responses of increased temperature, heart rate and sweating. In heat stroke, inflammatory response plays an important role and it is evidenced that turpentine (T) induced circulating inflammatory cytokines reduced survival rate and duration at 42°C. Here we report the alteration in the protein expression in liver cells upon HS with and without T treatment using two dimensional gel electrophoresis (2-DE), tryptic in-gel digestion and MALDI-TOF-MS/MS approaches

**Results:**

The effects of HS and T treatments alone and a combined treatments (T+HS) was performed in Wistar rat models. Proteomic analysis of liver in the HS and T+HS groups were analyzed compared to liver profiles of resting control and T treated groups. The study revealed a total of 25 and 29 differentially expressed proteins in the HS and T+HS groups respectively compared to resting control group. Fourteen proteins showed altered expression upon T treatment compared to resting control group. Proteins that are involved in metabolic and signal transduction pathways, defense, redox regulation, and cytoskeletal restructuring functions were identified. The altered expression of proteins reflected in 2D gels were corroborated by quantitative real time RT-PCR analysis of 8 protein coding genes representing metabolic and regulatory pathways for their expression and normalized with the house keeping gene β-actin

**Conclusion:**

The present study has identified a number of differentially expressed proteins in the liver cells of rats subjected to T, HS and T+HS treatments. Most of these proteins are implicated in cell metabolism, as well as adaptive response to incurred oxidative stress and tissue damage due to T+HS and HS effects.

## Background

Thermoregulation is a key physiological characteristic of humans and mammals. Exploration of the underlying mechanism of thermoregulation is of major concern to understand the patho-physiology of heat stress (HS) related illnesses. HS is induced by both exogenous and endogenous factors, and is associated with inflammatory and homeostatic responses [1]. HS results in responses of increased temperature, heart rate and sweating [2,3]. When exaggerated it can lead to heat stroke, a condition that involves a multitude of host-defense responses by activation of pro-inflammatory and inflammatory cytokines. Inflammatory response plays a significant role in the mechanistic pathways of HS lead stroke, which can cause clinical conditions of hemorrhage and multi-organ dysfunction [4,5].

The liver, as a major site of metabolism and detoxification, is a system of choice in studies involving toxicoproteomics, metabolic disorder and stress effects due to various pathobiological processes. It is evidenced the liver synthesizes acute phase proteins upon stimulation by cytokines that regulate physiologic response to inflammatory stimuli [6,7]. Previous studies have clearly demonstrated the effects of inflammatory cytokines involved in inflammation and associated pathological outcome of HS [8-10], and have utilized turpentine (T) administration as a method of choice for sterile induction of proinflammatory cytokines [11,12]. Although, these studies have provided a wealth of biochemical information on HS induced changes, early protein expression changes in the liver arising from the HS effect can be more characteristic and sensitive than pathological endpoints. We have previously investigated the effect of local inflammation induced by T treatment on the thermal effects of heat. T treatment compromised heat tolerance by enhancing the inflammatory response and tissue damage during heat stress. This is evident from decreased survival rate and duration at 42°C and elevated plasma cytokines IL-6, TNF-α and IL-1β [4]. Little is known about the cellular protein expression pattern of HS with and without T induced inflammation which could provide comprehensive data to understand the intrinsic pathways underlying the effect.

The study presented here examined the altered protein expressions in the liver of rats exposed to HS alone and with T treatment (T+HS). This was accomplished through a proteomic approach based on two-dimensional gel electrophoresis (2-DE) followed by in-gel tryptic digestion and MALDI-TOF-MS/MS for protein identification.

## Methods

### Materials

Immobilized pH gradient (IPG) strips (pH 3–10, 11 cm) and Criterion gels (10–20%, 4% stacking gel) for running 11 cm IPG strips were purchased from Bio-Rad (USA). CHCA (α-Cyano-4-hydroxycinnamic acid), ammonium bicarbonate, CHAPS and thiourea were purchased from Sigma Aldrich (St. Louis, MO, USA). Acetic acid, Acetonitrile (ACN) and trifluoroacetic acid (TFA) were from J. T. Baker (Griesheim, Germany). Mass spectrometry grade, Trypsin, was purchased from Promega Biosciences (San Luis Obispo, CA, USA). RNeasy^®^mini kit and RNase-free DNase-I were purchased from QIAGEN (USA). LightCycler FastStart DNA MasterPLUS SYBRGreen-I kit was obtained from Roche Diagnostics (Penzberg, Germany).

### Animal Experiments

Adult male Wistar rats (n = 24), weighing between 400 and 450 g were used. All animals were allowed to adapt to the environment for 1 week before the experiment and fed on laboratory chow. Water was provided *ad libitum*. In the conduct of animal experiments, we adhered to the guidelines for care and use of laboratory animals. All procedures received prior approval from the Institutional Animal Care and Use Committee for experimentation. Six rats were used per study group and subjected to heat stress (HS, T_c _42°C) with an infrared heating lamp positioned 40 cm above the rat. Tc was measured by a rectal probe inserted 8–10 cm past the anal sphincter and connected to a digital display for continuous monitoring. For turpentine treatment each group of rats (n = 6) treated with 0.05 ml turpentine (T) i.m. in the right hindlimb were compared with those of T and HS (T+HS). The volume of T and duration of passive treatment was based on our previous study data of IL-1β, IL-6 and TNF-α production 2 h post treatment [4].

### Two dimensional gel electrophoresis (2-DE)

2-DE was performed on treated and control samples with modification of the method as described [13]. To ensure reproducibility, all 2-DE gels were run under the same conditions and three gel replicates were used for each pooled rat liver extract to overcome inter-individual changes in animals. In brief, protein concentration of the lysate was adjusted to 200 μg/185μl volume of rehydration buffer and applied onto IPG strips (11 cm ReadyStrip IPG strips, Bio-Rad, USA). After16 h passive rehydration in the tray, first dimension isoelectric focusing (IEF) was run with the following programmed voltage parameters: 250 V for 1 h, 500 V for 1 h, 1000 V for 1 h, 2000 V for 1 h, 4000 V for 6 h, 500 V for 12 h, and 50 V for 5 h in Protean IEF cell (BioRad, USA) at 20°C. All gradients in voltage were programmed as linear gradients. Focused IPG strips was either subjected to a final 2D step or stored at -80°C until use. After equilibration of the strips the second-dimension SDS-PAGE was accomplished using Criterion precast gels (10–20%, 4% stacking gel, for use with 11 cm ReadyStrip IPG strips; 13.3 × 8.7 cm) and proteins visualized by silver staining protocol using Silver stain Plus™ stain as per manufacturer's instructions (Bio-Rad, USA). This method is highly sensitive to 1 ng level of detection with low background and is mass spectrometry compatible. The molecular masses were determined by running pre-stained standard protein markers (Precision plus protein standard kaleidoscope, Bio-Rad, USA) covering the range 10–250 kDa.

### Image analysis and tryptic in-gel digestion

Visual inspection of the gels was done to choose the best from the triplicates based on the highest image resolution and number of spots. The gels were then scanned and analyzed with PDQuest™ software (Bio-Rad, V7.2.0). Prominent gel spots were excised, reduced, and alkylated prior to digestion with 20 ng/μl of modified porcine trypsin. Extracted peptides were then desalted using Zip-Tip method (Agilent cleanup C18 Pipette Tips).

### MALDI-TOF/MS/MS analysis

For MALDI-TOF, 1 μl of the peptide mixture was spotted onto a metal MALDI-TOF target and allowed to air-dry. Thereafter, 1 μl of the CHCA matrix solution consisting of 10 mg/ml in 50% ACN and 0.1% TFA was applied to the dried sample and again allowed to air-dry. MALDI tandem mass spectrometry was performed on a TOF/TOF system (4800 Proteomics Analyzer, Applied Biosystems, USA). For MS mode, 800–4000 m/z mass range was used with 1000 laser shots per spectrum. A maximum of 10 precursors per spot with minimum signal/noise ratio of 50 were selected for MS/MS analysis. 1 KV collision energy was used for collision induced dissociation (CID), and 1500 acquisitions were accumulated for each MS/MS spectrum. Resultant spectra were searched against the International protein index (IPI) rat protein database from the European Bioinformatics Institute (EBI) using the GPS (Global Proteome Server) Explorer™ Software (v3.6, Applied Biosystems) running mascot search algorithm (v2.1, Matrix Science, London, UK) for protein identification. A mass tolerance of 100 ppm and 0.3 Da was used for precursors and fragment ions, respectively. MS/MS spectra were searched with GPS software using a 95% confidence interval (C.I.) threshold (p < 0.05), with which a minimum Mascot score of > 61 was considered imperative for further analysis. Representative protein coding genes (FAH, FBP1, ASS, DMGDH, PK, TKT, PPIA, and CEH) involved in metabolic and regulatory pathways were chosen for QRT-PCR confirmation of its differential expression pattern seen in 2D gels as analysed through PDQuest™ software. The expression levels were normalized with the house keeping gene β-actin

### Extraction of total RNA

Total RNA extraction was performed from rat liver extract of control and treated group using RNeasy^® ^mini kit. The RNA sample was subsequently treated with RNase-free Dnase-I at room temperature for 20 min and stored at -80°C until use. The quality and quantity of extracted RNA was determined by spectrophotometry (Bio-Rad, USA). All RNA samples used for Quantitative fluorescence-based real-time PCR (QRT-PCR) experiments were of highest purity with A_260_/A_280 _ratios of 1.9–2.1.

### QRT-PCR confirmation of representative genes for their differential expression

QRT-PCR was carried out according to the manufacturer's instructions using the LightCycler system (Roche Diagnostics, Penzberg, Germany) as an independent method for assessing the relative gene expression. A set of 8 genes (coding proteins FAH, FBP1, ASS, DMGDH, PK, TKT, PPIA, and CEH) were analyzed for their expression patterns. Reverse Transcription (RT) of RNA and QRT-PCR were performed as follows: single stranded cDNA was generated from 3 mg of total RNA, 200 mM nucleotides, 500 units Superscript II reverse transcriptase (Invitrogen) and 1.5 mM oligo(dT)_15 _primers in 50 μl reactions. Reverse transcription was stopped after 1 h by heating to 95°C for 5 min. The sense and antisense primers used for specific amplification are shown in Table 1. The primers were designed and subsequently checked for specificity using NCBI-BLAST. All primers were synthesized by 1st BASE Pvt Ltd. (Singapore). The expression of β-actin was used as an internal calibrator for equal RNA loading, and to normalize relative expression data for all other genes analyzed. The copy ratio of each analyzed cDNA was determined as the mean of three experiments and quantified using the relative quantification (2^-ΔΔCT^) method [14].

**Table 1:** Primer sequences used in quantitative real-time RT-PCR analysis of rat liver extracts

**NCBI Acc**	**Gene Annotation**	**Gene name**	**primer sequence**	**Product size (bp)**
8393349	fumarylacetoacetate hydrolase	FAH	5'-gccagccctacacatttgat-3' 5'-tcagttgctgcagaatggtc-3'	123
50926831	fructose-1,6-biphosphatase1	FBP1	5'-tggaccccatgataactggt-3'	123
8394009	peptidylprolyl isomerase A	PPIA	5'-agcactggggagaaaggatt-3' 5'-ttcaccttcccaaagaccac-3'	281
56689	dimethylglycine dehydrogenase	DMGDH	5'-attgacctgtctccgtttgg-3' 5'-attggtgggaaacagtcagc-3'	166
12018252	Transketolase	TKT	5'-gccaggttcagagcaaaaag-3' 5'-atccataggctttccgtgtg-3'	141
40363265	pyruvate kinase, liver and RBC	PK	5'-agtcggaggtggaaattgtg-3' 5'-acccgggtgatattgtggta-3'	115
15459757	carboxylic ester hydrolase	CEH	5'-ggtctggctttttcttgctg-3' 5'-ccaacagcatcttgagagca-3'	261
25453414	arginosuccinate synthetase	ASS	5'-ccatcctttaccacgctcat-3' 5'-Tgctcaccagctcttcattg-3'	267
42475962	β-actin (house keeping gene)		5'-tcatgaagtgtgacgttgacatccgt-3' 5'-cctagaagcatttgcggtgcaggatg-3'	285

### Statistical analysis

The unpaired t-test and one-way ANOVA was used to compare the relative ratio of gene expression among the treatment groups. Statistical analyses were performed using Graphpad Prism version 4.01 for Windows, Graphpad software. Results are represented as mean ± standard error of the mean (SEM) with P level of < 0.05 significance.

## Results and Discussion

**Figure 1:**
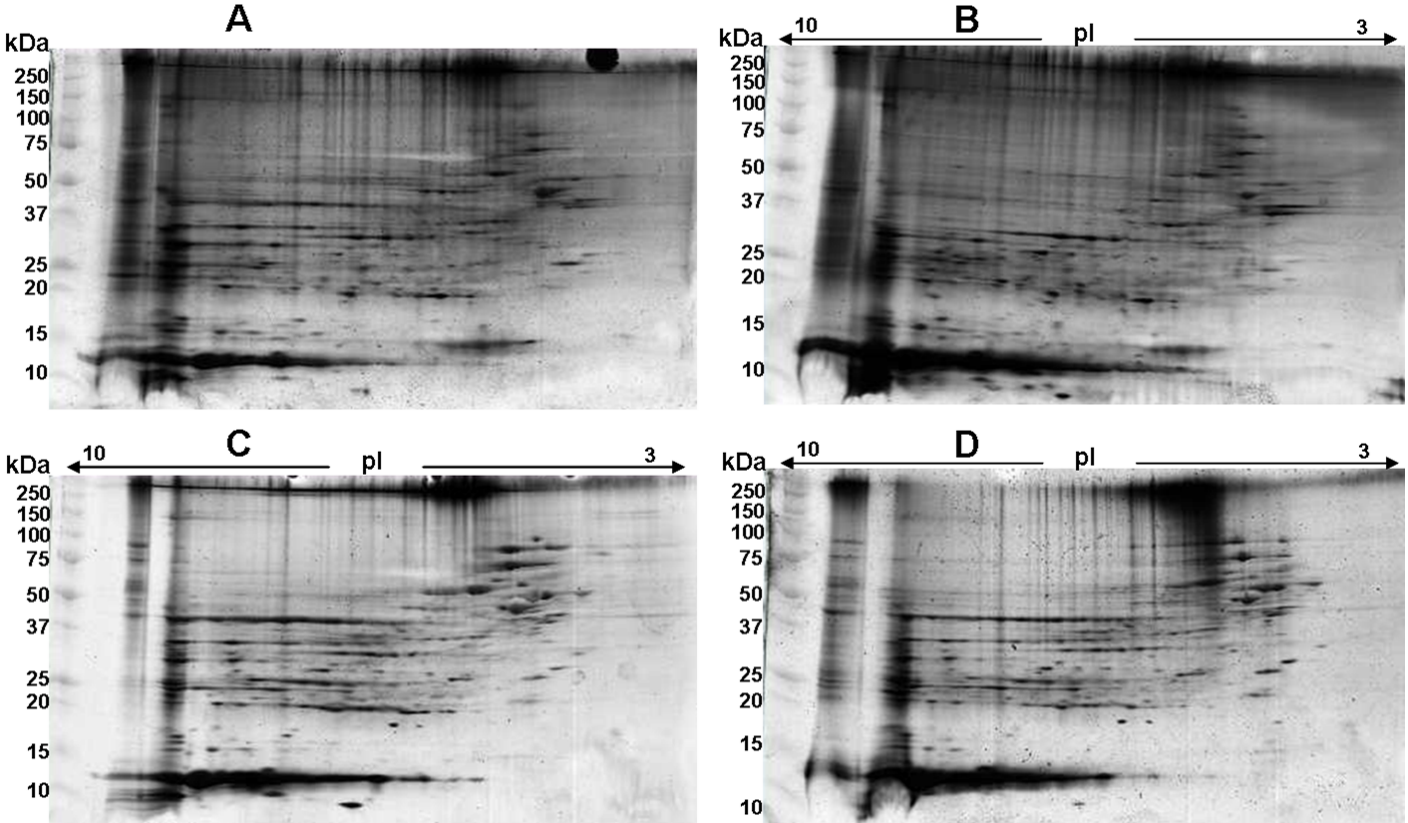
**Representative image of 2D gels (silver stained)**. Rat liver protein profile analyzed by 2D-GE of (A) Control (T = 37°C) group (B) Heat stress (HS, T = 42°C) group (C) turpentine (T) treatment group and (D) T with HS (T+HS) group. First dimension performed using immobilized pH 3–10 gradient strips, followed by SDS-PAGE in 10–20% polyacrylamide gels.

2-DE of rat liver extract of treatment groups (HS, T, and T+HS)have identified a number of differentially expressed proteins compared to the control group as examined by scanning and image analysis of silver stained gels (figure 1). MALDI-TOF/MS/MS data analysis using MASCOT identified a number of GenBank annotated proteins under each condition that were then curated, and functional roles analyzed using pathway analysis software (Ingenuity Pathway Analysis, USA). The study revealed proteins of different functions including signal transduction pathways, cell and energy metabolism, defense, apoptosis, and respiration. A list of these proteins and their properties can be found in the additional file 1. 2-DE coupled with MS is currently the key approach for profiling thousands of proteins of a given proteome simultaneously and offer the possibility to analyze complex biological processes directly on the level of quantitative protein expression. However, this method is labour-intensive and not readily interfaced with protein identification by MS. In addition hydrophobic, extremely basic proteins and those with a very high or low MW are often difficult to analyse using this approach

Fourteen proteins showed altered expression upon turpentine treatment without HS, 6 up-regulated and 8 down-regulated as shown (Table 2). Twenty-five differentially expressed proteins (including 21 up-regulated and 4 down-regulated) in the HS group and 29 (22 up-regulated and 7 down-regulated) in the T+HS group were identified compared to resting control group. Proteins that are up-regulated in T+HS compared to T group include Tubulin beta, Aldehyde dehydrogenase (ADH), Sorbitol dehydrogenase (SDH), L-iditol-2-dehydrogenase, fructose-1,6-biphosphatase1 (FBP1), pyruvate kinase (PK)and carboxylic ester hydrolase (CEH). Down-regulated proteins in T+HS group compared to T group include Liver glycogen phosphorylase isozyme, eukaryotic translation elongation factor-2 and H(+)-transporting ATP synthase. A total of 8 protein coding genes (*Ceh, Ppia, Pk, Tkt, Dmgdh, Ass, Fah*, and *Fbp1*) were chosen based on their consistency in the 2D gels and their representing roles in metabolism (carbohydrate, aminoacid) and biological regulation (hydrolase, transketolase activities) for quantitative expression analysis. A quantitative real-time RT-PCR analysis was used to determine the gene expressions in the HS and T+HS groups and compared against control using the relative quantification (2^-ΔΔCT^) method. The expression level of each gene was normalized to the house keeping gene, β-actin. Using a cutoff value of ≥ ± 2-fold change in transcript abundance in all the experiments, a total of 7 genes (5 increased and 2 decreased significantly, P < 0.05) among the HS group and 6 genes (3 increased and 3 decreased, P < 0.05) among the T+HS group have revealed significant changes in their expression (Table 3).

**Table 2:** List of altered protein expression upon turpentine (T) treatment

**Down-regulated proteins**	**Up-regulated proteins**
Mitochondiral aldehyde dehydrogenase precursor	Liver glycogen phosphorylase isozyme
Mitochondiral aldehyde dehydrogenase	Alcohol dehydrogenase
Aldehyde dehydroganase 2	Peptidylprolyl isomerase A
Tubulin, beta 5	Microsomal GST 1
Glutathione-S-Transferase (GST), Mu type 3	Eukaryotic translation elongation factor 2
GST, alpha type 2	III beta-2-globin
Ribosomal protein S19	
Hist2haa1 protein	

**Table 3:** The expression pattern of genes as analyzed by Quantitative real time RT-PCR

**Gene**	**HS group**			**T+HS group**		
	
	**Mean fold changes (2^-ΔΔCT^)^c^**	**SD^a^**	**CV^b^**	**Mean fold changes (2^-ΔΔCT^)^c^**	**SD**	**CV**
FAH	-4.55*	0.76	16.76	-11.18*	1.27	11.34
FBP1	-1.06	0.05	4.72	-1.23	0.15	12.14
ASS	-2.21*	0.33	15.12	-2.07*	0.41	19.74
DMGDH	2.73*	0.57	20.95	-2.99*	0.69	23.31
PK	10.12*	0.81	8.02	19.11*	2.18	11.4
TKT	4.67*	0.66	14.13	-1.30	0.30	22.96
PPIA	18.01*	2.21	12.27	3.02*	0.64	21.06
CEH	34.54*	7.92	22.94	4.69*	0.65	13.90

The fold changes are mean of three values compared to control groups and using a cut-off of ≥ ± 2.0 for significance (*P < 0.05).

(a) The standard deviation (SD) quantifies variability or scatter of a data set, and is expressed in the same units as the data. If the data are sampled from a Gaussian distribution, then 95% of the values is expected to lie within two SD of the mean.

(b) Coefficient of variation (CV) represents the ratio of the SD to the mean of the data and indicates the relative variability of SD value to the mean value of the datasets.

(c) The samples were analyzed using QRT-PCR and the C_t _data were imported into Microsoft excel. The mean fold change in expression of the target gene at HS and T+HS was normalized relative to the house keeping gene β-actin and calculated by the formula **2^-ΔΔCT^**, where **ΔΔ**C_T _= (C_T Target _– C_T Actin_) treated – (C_T Target _– C_T Actin_) control. The mean ± SD values of β-actin calculated for control, HS and T+HS were 18.72 ± 0.516, 24.04 ± 0.106 and 25.78 ± 0.092 respectively.

Prominent proteins that are down-regulated both in the HS and T+HS groups include FAH and ASS (Table 3). Significant suppression of FAH (P < 0.01) was observed in T+HS group compared to control and HS groups. FAH is the last enzyme involved in the breakdown of tyrosine, and suppression of this enzyme would eventually lead to accumulation of upstream metabolites such as succinylacetone, maleylacetoacetate (MAA), and fumarylacetoacetate (FAA). FAA is cytotoxic to cells and leads to cell cycle arrest and apoptosis. It also causes Golgi apparatus disruption and elicits ER stress response [15]. This is elucidated by the fact that with their electrophilic properties, MAA and FAA are likely to interfere with sulfhydryl reactions by forming adducts with glutathione and other sulfhydryl groups [16]. Downregulation of ASS may interfere with the cellular regulation of nitric oxide (NO), caspase 3 (CASP3), arginine and Bcl2. T+HS group has revealed pronounced cellular perturbances in the liver compared to the HS group.

Dimethylglycine dehydrogenase (DMGDH) found with increased expression in the HS group, on the contrary, was down-regulated (-2.99, P < 0.05) among the T+HS group. Down-regulation of this mitochondrial enzyme could eventually affect the metabolic fate of choline by preventing catalysis of oxidative demethylation of dimethylglycine (DMG) to sarcosine and ultimately glycine. Deficiency of DMGDH is involved in the accumulation of DMG in the urine and serum of patient who presented with "fish-odor syndrome". This appeared to be the result of a homozygous point mutation in the DMGDH gene [17].

Substantial up-regulation of PPIA and CEH were observed in the HS group compared to the T+HS (P < 0.01) group (Table 3). CEH catalyzes the hydrolysis of various xenobiotics and endogenous substrates such as ester, amide, and thioester bonds and are thought to function mainly in drug metabolism [18]. Overexpression of CEH (fold change 34.54) and chaperone protein PPIA (18.01) in the HS group could probably indicate an adaptive response of liver to sustain cell homeostasis from thermal injury. Despite its role in facilitating protein folding and assembly in response to a cellular stress, the induced expression of PPIA exert a protective effect helping to cope with insults and to prevent apoptosis [19].

Pyruvate kinase (PK) is a hydrolase enzyme involved in glycolysis and the elevated expression of this enzyme in the HS (10.12 fold) and T+HS (19.11 fold) groups (Table 3) revealed acute alterations in energy metabolism to maintain cell homeostasis. This is in agreement with a previous study on glial cells in which up-regulation of PK was found associated with tolerance to hypoxia induced ischemic stress [20]. Transketolase (TKT) is a thiamine dependent enzyme that plays critical roles in the providing key phosphorylated carbohydrate intermediates to the cell and thereby connects the PPP to glycolysis [20,21]. TKT (protein score 87) was significantly up-regulated in HS group (fold change 4.67, P < 0.01) and no significant variation was noticed among the T+HS group (-1.30, P < 0.05 not significant). Differential expression of TKT may have an effect on the PPP (pentose phosphate pathway) metabolic pathway, besides methyltransferase and oxidoreductase activities [22]. The study result coincides with a study report wherein the elevated TKT expression was related to activation of the oxidative branch of the PPP and augmented cellular defense against oxidative damage [23].

Other notable proteins that are up-regulated in the T+HS group include phosphatidylethanolamine binding protein (PEBP1, protein score 108), glutathione S-transferase (GST)-Mu1 (protein score 506), urate oxidase (UOX, score 155), sorbitol dehydrogenase (SDH, score 246), tumor rejection antigen gp96 (score 217) and apolipoprotein-E (score 186). PEBP1, a cytosolic protein also known as "Raf-1 kinase interacting protein" (RKIP), has been shown to be differentially expressed in the disease process of acute liver failure (ALF) and hypothesized as a potential plasma biomarker for ALF diagnosis [24]. Increased GSTs in the present study is indicative of the presence of increased lipid peroxides in the T+HS treated rat livers. GSTs have been shown to play role in detoxification of toxic substances, xenobiotics, and products of oxidative stress [25]. Elevated UOX (protein score 155) and SDH (protein score 246) expression maybe in response to disruption caused in the liver. In support of the data, the expression level of SDH was found directly proportional to the extent of liver damage [26]. Gp96 is a member of the Hsp90 family of molecular chaperones that resides within the lumen of endoplasmic reticulum (ER). In addition to its chaperone role, administration of tumor derived gp96 induces protective tumor-specific immunity and anti-inflammatory properties [27-29]. Up regulation of gp96 (protein score 217) in the T+HS group is apparently an adaptive response to induced inflammation.

In the HS group enoyl coenzyme A-hydratase (ECHS), aldehyde dehydrogenase (ALDH1), laminin receptor (LR)-1 and betaine-homocysteine methytransferase (BHMT) were up-regulated (for details refer additional file 1). BHMT is involved in the pathway of choline oxidation. It catalyzes a methyl transfer from betaine to homocysteine to form dimethylglycine and methionine, respectively [21,30,31]. Up-regulation of BHMT could be a protective response to reduce the generated oxidative stress in liver. On the other hand, decreased BHMT has eventually led to the impairment of mitochondrial function and generation of oxidative stress [30-34]. Laminin is a cell adhesion protein important for development and maintenance of specialized tissue architecture. In the adult rat liver, it is present in small amounts as part of an incomplete basement membrane separating endothelial cells of the sinusoids from hepatocytes [35]. We observed an up-regulation of LR-1 in liver cells of HS rats compared to resting controls. On the other hand, a proteomic studies on rat liver have not identified the LR-1 among the 273 proteins reported. For more details please refer Table 1 of the article cited [36] is indicative of an adaptive response to maintain the integrity of the cells.

A report on proteome analysis of liver or rats with chronic alcohol consumption that induce oxidative stress and elevate proinflammatory cytokines has identified changes in abundance of proteins. The study has revealed up-regulation of UOX, FAA, ECHS and aldolase B, and down-regulation of FBP1, TKT, ETF, HSP90, enolase, and GAPDH [37]. We do observe some similarities and disparity in the protein expression profiles compared to their study. Similarities in protein pattern include an up-regulation of UOX and FAA, and down-regulation of TKT, was observed in the T treatment group. On the contrary, ECHS was down-regulated, and ETF, enolase and HSP90 were up-regulated. Aldolase B and GST mu type 2 expression remain normal in our study

A recent study has demonstrated that in rats, during a localized inflammation induced by intramuscular T administration, the expression of heme-oxygenase-1 (HO-1) is strongly induced in the macrophages of the injured muscle and in liver parenchyma. HO-1 play important role in cytoprotection of hepatocytes, which might become damaged under inflammatory conditions [38]. In contrast, we have not observed differential expression of HO-1 upon T treatment in our study.

## Conclusion

In summary, we performed the proteome analysis of rat liver under the conditions of HS and induced inflammation with HS (T+HS). Comparison of 2- DE patterns of the control group with those of treatment groups has identified a number of differentially expressed proteins involved in energy metabolism and signal transduction pathways. Significant alterations in the expression of PK, ASS, DMGDH, PPIA, CEH, TKT and FAH have important implications in the mechanistic pathways of metabolism and regulation induced tissue damage and inflammation. PEBP1, SDH, GSTs, BHMT, UOX, and ECHS1 are other prominent proteins, the participation of which in metabolism reflects the importance in the regulation of redox equilibrium. Further work will be necessary to measure these up-regulated proteins and will allow a better comprehension of the cellular events underlying the effect.

## Competing interests

The authors declare that they have no competing interests.

## Authors' contributions

RG, LKW and SP performed protein extraction, two-dimensional gel electrophoresis, identification of differentially expressed proteins, and data handling, LCL did the animal experiments, TK did the MALDI-TOF-MS/MS analysis, GPs' facility confirmed the differential expression of genes by QRT-PCR, YEP, LJ and MS designed the study.

## Supplementary Material

Additional file 1 **Supplementary table.** Prominent annotated proteins that exhibited differential expression upon treatment as identified by MALDI-TOF-MS/MS. Click here for file
