# Supplementary material for: A study on the differential protein profiles in liver cells of heat stress rats with and without turpentine treatment
Source: Proteome Sci. 2009 Jan 7;7:1. doi: 10.1186/1477-5956-7-1 (PMC2626589; doi:10.1186/1477-5956-7-1)
Supplement: Additional file 1 — Supplementary table. Prominent annotated proteins that exhibited differential expression upon treatment as identified by MALDI-TOF-MS/MS. [file 1477-5956-7-1-S1.doc]

## Prominent annotated proteins that exhibited differential expression upon treatment as identified by MALDI-TOF-MS/MS

| **Condition** | ***i*Pro Class Number^a^** | **Accession Number (GI)^b^** | **Protein Name** | **pI** | **M_r_ (kDa)** | **Protein**  **score^c^** | **Function / Process** |
| --- | --- | --- | --- | --- | --- | --- | --- |
| **Carbohydrate, lipid, aminoacid and energy metabolism** | | | | | | | |
| HS (↑) | P14141 | 31377484 | Carbonic anhydrase 3 | 6.89 | 296.9 | 101 | Ion binding and lyase activity; 1-C compound metabolic process |
| HS (↓), T+HS (↓) | P19112 | 50926831 | Fructose-1,6-bisphosphatase 1 | 5.54 | 39.6 | 179 | Catalytic, hydrolase and ion binding activities; carbohydrate, alcohol and organic acid metabolism. Important role in PPP, Glycolysis/ gluconeogenesis |
| HS (↑) | P09811 | 11560087 | Liver glycogen phosphorylase | 6.75 | 97.5 | 83 | Catalytic and transferase activity, and nt, vitamin and cofactor binding; carbohydrate, polysaccharide, and energy reserve metabolic processes. Role in biosynthesis of phosphoribosyl pyrophosphate and glycogen metabolism |
| HS (↑) | P04797 | 56611127 | Glyceraldehyde-3-phosphate dehydrogenase (GAPDH) | 8.14 | 36.1 | 75 | Oxidoreductase, protein and cofactor binding activities; carbohydrate, alcohol, macromolecule metabolic processes, developmental process |
| T+HS (↑) | Q5EB49 | 59808815 | Enolase 1, alpha | 6.16 | 47.5 | 145 | Ion binding and lyase activities; carbohydrate, alcohol and macromolecule metabolic processes. |
| T+HS (↑) | P16617 | 40254752 | Phosphoglycerate kinase 1 | 8.02 | 449.1 | 73 | Nt binding, transferase activity; carbohydrate, alcohol, macromolecule and phosphorous metabolism |
| HS (↓), T+HS (↓) | P09034 | 25453414 | Arginosuccinate synthase | 7.63 | 46.8 | 50 | Nt binding and ligase activity; aminoacid metabolism |
| HS (↑), T+HS (↓) | Q63342 | 56689 | Dimethylglycine dehydrogenase, mitochondrial precursor | 6.91 | 96.1 | 69 | Nt and cofactor binding, and transferase and oxidoreductase activities; aminoacid metabolism, electron transport. Catabolism of choline. |
| HS (↑) | P11884 | 45737866 | mitochondrial aldehyde dehydrogenase precursor | 6.63 | 56.5 | 102 | Oxidoreductase activity; response to stimulus, metabolic process |
| T(↑) | P06757 | 202737 | Alcohol dehydrogenase | 8.52 | 396.4 | 73 | Oxidoreductase and ion binding activity; catalyzes reactions involving NAD+, NADH; cellular lipid metabolism, and play role in glycolysis / gluconeogenesis |
| HS (↓), T+HS (↓) | P25093 | 8393349 | Fumarylacetoacetate hydrolase | 6.67 | 45.9 | 102 | Catalytic, hydrolase and ion binding activities, involved in aminoacid (tyrosine) and organic acid metabolism |
| HS (↑), T+HS (↑) | P12928 | 40363265 | Pyruvate kinase, liver and RBC | 6.45 | 62.2 | 175 | Hydrolase enzyme involved in glycolysis, play role in ion binding and transferase activity; carbohydrate, alcohol, and macromolecule metabolic processes, response to stimulus |
| **Defense** | | | | | | | |
| HS (↑) | O09171 | 13540663 | Betaine-homocysteine methyltransferase 2 | 8.02 | 45.4 | 52 | Transferase and ion binding activities; hepatic methionine metabolism, biological regulation and protein modification processes |
| T+HS (↑) | P04905 | 204503 | Glutathione S-transferase Yb-1 subunit (Mu 1) | 7.63 | 26.1 | 506 | Steroid binding, transferase activity; metabolic, neurological system process and response to stimulus |
| T+HS (↑) | P04904 | 13928688 | glutathione S-transferase, A5 | 8.78 | 25.4 | 149 | Transferase activity; metabolic process and response to stimulus |
| T+HS (↑) | P00502 | 51036637 | glutathione S-transferase, alpha type1 | 8.87 | 25.6 | 91 | Transferase activity; metabolic process |
| T+HS (↑) | P08009 | 13592152 | glutathione S-transferase, Yb-3 (Mu type3 | 6.84 | 25.7 | 96 | Transferase activity; metabolic process |
| **Respiration** | | | | | | | |
| HS (↑) | P02091 | 984679 | Beta-globin | 7.88 | 16.1 | 72 | Ion and oxygen binding, and transporter activities; gas transport |
| HS (↑) | P02091 | 17985949 | Hemoglobin beta chain complex | 7.88 | 16.1 | 100 | Ion and oxygen binding and transporter activities; gas transport |
| HS (↑) | P01946 | 30027750 | Alpha-globin | 6.49 | 94.03 | 109 | Bind ion, oxygen and tetrapyrrole, transporter activity; gas transport |
| **Signal transduction** | | | | | | | |
| T+HS (↑) | Q68FU3 | 5148412 | Electron-transfer-flavoprotein (ETF), beta polypeptide | 7.6 | 278.9 | 134 | Electron transport. Transfers electrons to the mt respiratory chain via ETF-dehydrogenase |
| HS (↑), T+HS (↓) | P14604 | 40555865 | Enoyl Coenzyme A hydratase, short chain, 1, mitochondrial | 8.4 | 31.5 | 67 | Catalytic and lyase activities; lipid and organic acid metabolism; role in biodegradation of xenobiotics and biosynthesis of secondary metabolites (Limonene/pinene degradation) |
| T+HS (↑) | P31044 | 8393910 | Phosphatidylethanolamine binding protein (PEBP-1) | 5.48 | 209 | 108 | Nt and lipid binding activities, and enzyme regulator |
| **Redox regulation** | | | | | | | |
| HS (↑) | P31399 | 220904 | ATP-synthase, H+transporting, mitochondrial F0 complex, subunit d. | 5.78 | 18.8 | 74 | Transporter and hydrolase activities; oxidative phosphorylation, ion, hydrogen transport ant nt metabolic processes |
| T+HS (↑) | P15999 | 40538742 | ATP synthase, H+ transporting, mitochondrial F1 complex, alpha subunit, isoform 1 | 9.22 | 59.8 | 57 | Transporter and hydrolase, and ion binding activities; oxidative phosphorylation, ion transport nt metabolism |
| T+HS (↑) | O35244 | 16758348 | peroxiredoxin 6 | 5.64 | 24.8 | 94 | Catalytic, hydrolase, antioxidant and oxidoreductase activities; lipid metabolism and response to stimulus |
| **Apoptosis** | | | | | | | |
| HS (↑) | P05197 | 8393296 | eukaryotic translation elongation factor 2 | 6.41 | 95.3 | 44 | Hydrolase, nt binding, and translation regulator activities; translation and biopolymer process. GTP-dependent translocation of nascent protein chain |
| **Cytoskeletal restructuring** | | | | | | | |
| HS (↓), T+HS (↑) | P69897 | 27465535 | Tubulin, beta 5 | 4.78 | 49.7 | 66 | Nt binding, hydrolase and structural molecule activities; protein polymerization, cellular component organization and transport processes |
| **Others** | | | | | | | |
| HS (↑) | P16303 | 140969642 | Carboxylesterase 3 precursor | 6.1 | 62.2 | 76 | Hydrolase activity, role in lipid and xenobiotic metabolism |
| HS (↑) | P06761 | 25742763 | Heat shock 70kD protein 5 (GRP78) | 5.07 | 72.5 | 377 | Binds protein, nt, rnp, and enzyme regulator activity. Cellular and developmental process and biological regulation |
| HS (↑) | P06761 | 554440 | glucose-regulated protein, 78kDa precursor (GRP78) | 5.07 | 72.4 | 90 | Protein, nt, RNP binding, enzyme regulator activities; Cell communication, biological regulation and developmental processes and response to stimulus |
| HS (↑) | P38983 | 8393693 | laminin receptor 1 | 4.8 | 32.9 | 106 | Structural molecule activity; translation process |
| HS (↑) | Q6P7R7 | 13928952 | UBA52 (Ubiquitin A-52 residue ribosomal protein fusion product 1) | 9.87 | 14.7 | 262 | Structural molecule activity; translation and protein modification process, role in membrane trafficking and cell signalling |
| HS (↑) | P62989 | 20302085 | ubiquitin | 6.56 | 8.6 | 231 | Protein modification process |
| HS (↑), T+HS (↑) | Q91YG2 | 15459757 | Carboxylic ester hydrolase | 6.10 | 62.1 | 74 | Hydrolase activity |
| HS (↓), T+HS (↑) | P08011 | 19705453 | microsomal glutathione S-transferase 1 | 9.62 | 17.5 | 96 | Transferase activity; cofactor metabolic process, conjugates GSH to a wide number of hydrophobic electrophiles. |
| HS (↑), T+HS (↑) | P10111 | 8394009 | Peptidyl-prolyl isomerase A | 8.34 | 18.1 | 101 | Isomerase and protein binding activities; protein folding, biological regulation. Role in cis-trans isomerization of peptidylproline peptide bonds. |
| HS (↑), T+HS (↓) | P50137 | 12018252 | Transketolase | 7.22 | 67.6 | 87 | Transferase and ion binding activities; biological regulation. Role in calcium ion binding |
| T+HS (↑) | Q66HD0 | 58865966 | Tumor rejection antigen gp96 (predicted) | 5.02 | 74.4 | 217 | Nt and protein binding; protein folding and response to stimulus |
| T+HS (↑) | P63039 | 51702230 | 60kDa HSP, mitochondrial precursor | 5.91 | 61.1 | 156 | Nucleotide (nt) and protein binding; involved in protein folding |
| T+HS (↑) | P17988 | 55765 | Aryl sulfotransferase | 6.41 | 33.4 | 235 | Transferase activity and nt binding; lipid and drug metabolism |
| T+HS (↑) | Q6PAH0 | 37805241 | Apolipoprotein E | 5.23 | 35.8 | 186 | Transporter activity and lipid binding; protein metabolism and lipid transport |
| T+HS (↑) | UPI00001819EF | 62662278 | Similar to GAPDH | 8.09 | 36.3 | 71 | - |
| T+HS (↑) | P27867 | 77404286 | Sorbitol dehydrogenase (L-iditol 2-dehydrogenase) | 7.14 | 38.2 | 246 | Oxidoreductase activity, ion and cofactor binding. Role in fructose and mannose metabolism, catalyzes reaction involving H+, NAD+, NADH, D-fructose, L-iditol, sorbitol. |
| T+HS (↑) | P34058 | 51859516 | Heat shock 90kDa protein 1, beta | 4.97 | 83.3 | 74 | Nt and protein binding activities; protein folding and response to stimulus |
| T+HS (↑) | P82995 | 28467005 | Heat shock protein (HSP) 90- 1, alpha | 4.93 | 85.2 | 101 | Nt and protein binding activities; protein folding and response to stimulus |
| T+HS (↑) | P63018 | 13242237 | HSP 8 (heat shock 70 kDa protein 8) | 5.37 | 71.1 | 119 | Nt binding; protein folding and response to stimulus |
| T+HS (↑) | P09118 | 137108 | Uricase (Urate oxidase) | 8.2 | 35.1 | 155 | Oxidoreductase activity; nt, aromatic compound and heterocycle metabolic processes |

# ↑ - indicate up-regulation; ↓ - indicate down-regulation

(a) iProClass is an integrated protein knowledgebase of protein information resource (PIR) database, provides value-added information reports for UniProtKB and unique NCBI Entrez protein sequences in UniParc, with links to over 90 biological databases, including dB for protein families, functions and pathways, interactions, structures and structural classifications, genes and genomes, ontologies, literature, and taxonomy

(b) GI number is simply a series of digits that are assigned consecutively to each sequence record processed by the NCBI. The GI system of sequence identifiers runs parallel to the accession-version system which was implemented by GenBank, EMBL & DDBJ in February 1999

(c) Score is -10*Log(P), where P is the probability that the observed match is a random event, based on the NCBInr database using the MASCOT searching program as MS/MS data.
